# Suggested Mechanisms of Tracheal Occlusion Mediated Accelerated Fetal Lung Growth: A Case for Heterogeneous Topological Zones

**DOI:** 10.3389/fped.2017.00295

**Published:** 2018-01-12

**Authors:** Ahmed I. Marwan, Uladzimir Shabeka, Evgenia Dobrinskikh

**Affiliations:** ^1^Division of Pediatric Surgery, Department of Surgery, University of Colorado Denver School of Medicine, Denver, CO, United States; ^2^Department of Medicine, University of Colorado Denver School of Medicine, Denver, CO, United States

**Keywords:** heterogeneous topological zones, pulmonary hypoplasia, tracheal occlusion, lung growth, fetal surgery, noise and order

## Abstract

In this article, we report an up-to-date summary on tracheal occlusion (TO) as an approach to drive accelerated lung growth and strive to review the different maternal- and fetal-derived local and systemic signals and mechanisms that may play a significant biological role in lung growth and formation of heterogeneous topological zones following TO. Pulmonary hypoplasia is a condition whereby branching morphogenesis and embryonic pulmonary vascular development are globally affected and is classically seen in congenital diaphragmatic hernia. TO is an innovative approach aimed at driving accelerated lung growth in the most severe forms of diaphragmatic hernia and has been shown to result in improved neonatal outcomes. Currently, most research on mechanisms of TO-induced lung growth is focused on mechanical forces and is viewed from the perspective of homogeneous changes within the lung. We suggest that the key principle in understanding changes in fetal lungs after TO is taking into account formation of unique variable topological zones. Following TO, fetal lungs might temporarily look like a dynamically changing topologic mosaic with varying proliferation rates, dissimilar scale of vasculogenesis, diverse patterns of lung tissue damage, variable metabolic landscape, and different structures. The reasons for this dynamic topological mosaic pattern may include distinct degree of increased hydrostatic pressure in different parts of the lung, dissimilar degree of tissue stress/damage and responses to this damage, and incomparable patterns of altered lung zones with variable response to systemic maternal and fetal factors, among others. The local interaction between these factors and their accompanying processes in addition to the potential role of other systemic factors might lead to formation of a common vector of biological response unique to each zone. The study of the interaction between various networks formed after TO (action of mechanical forces, activation of mucosal mast cells, production and secretion of damage-associated molecular pattern substances, low-grade local pulmonary inflammation, and cardiac contraction-induced periodic agitation of lung tissue, among others) will bring us closer to an appreciation of the biological phenomenon of topological heterogeneity within the fetal lungs.

## Introduction

“One of the basic principles of life is ‘organization’ by which we mean that if two things are put together something new is born, the qualities of which are not additive and cannot be expressed in terms of the qualities of the constituents.” *[Albert Szent-Gyorgyi. Introduction to a Submolecular Biology. P.11 (New York: Academic Press, 1960)]*.

A fundamental feature of living biological systems is a stable function overlying an existing background of constantly competing excess external and internal signals. How such “chaos” converts into “order” with fine tuning of the function of living systems is still an enigma. Development of fetal lungs is a classic example of how a “chaos” of biological signals ultimately converts in a precise time-specific pattern to a flawless harmony. It is a fascinating example of perfect interplay between systems. A uniquely complicated picture is seen in the setting of accelerated lung growth following fetal tracheal occlusion (TO). Occluding the trachea during pregnancy has been offered to the most severe forms of CDH to augment lung growth. Such approach is based on animal models of tracheal agenesis demonstrating lung growth in the affected fetuses.

What are the biological signals and mechanisms involved in accelerated lung growth following TO and how do these signals and mechanisms coordinate and interact?

Furthermore, does the interaction of these different signals induced by TO change postnatal pulmonary development, structure and function and does it produce a uniform homogeneous background in the fetal lungs? The answers to these questions are currently unknown.

## Lung Development, CDH, and Effects of TO

### Embryonic Lung Development

The main task of the lungs is to provide gas exchange to sustain life. During fetal development, the lung forms as a liquid-filled organ with its many integrated cellular types and structures. It involves an astonishing and precisely orchestrated series of molecular, cellular, and tissue events. A detailed review of normal lung development is beyond the scope of this article and the reader is referred to several excellent recent publications (1–4).

Briefly, lung development spans an overlapping five stages starting early during the embryonic phase of development and ending in early childhood. The initial lung primordium is seen at the fifth week of gestation as an evagination from the foregut and subsequently forms the right and left lung buds with their main bronchi. Branching morphogenesis subsequently commences during the pseudoglandular stage of lung development to form a branching network of communicating conducting airways. Fetal breathing movements (FBMs) are seen toward the end of this stage (8 weeks) and will result in mechanical forces applied to the developing lungs (5). Ongoing development ultimately results in differentiation of the airway epithelial cells during the canalicular stage. Type I and type II epithelial cells are seen marking the morphological differentiation between conducting and respiratory airways and the start of surfactant production (6). In addition, during this stage, the pulmonary acinus, which is the basic functional pulmonary ventilatory unit, is identified (7). At the end of the canalicular stage, gas exchange may take place and, thus, this represents the cutoff for human premature neonatal survival. The hallmarks of the saccular stage include remodeling of the acinus with thinning of the connective tissue septa and further maturation of the surfactant machinery preparing the baby for *ex-utero* life. Finally, during the alveolar stage that extends to 36 months postnatally, progressive secondary septation occurs associated with remodeling of the pulmonary vascular tree (8).

### Pulmonary Vascular Development

Embryonic pulmonary vascular development entails two distinct, yet interconnected mechanisms; vasculogenesis, which is the *de novo* formation of new blood vessels from endothelial progenitor cells and is thought to be responsible for the formation of the peripheral pulmonary circulation (9, 10) and angiogenesis which entails new vessel sprouting from pre-existing ones, follows the axis of the developing large airways and is responsible for the formation of the proximal pulmonary vessels. In the lung, vasculogenesis and angiogenesis occur concomitantly with branching morphogenesis endorsing a cross talk between pulmonary vascular development and lung organogenesis (11). During the pseudoglandular stage, the proximal and distal vessels begin to join to form the complete pulmonary circulation, which continues to follow the branching pattern of the developing airways. By the end of the pseudoglandular stage, all preacinar pulmonary and bronchial arteries are formed and correspond to the branching pattern of the conducting airways (1).

### Clinical Relevance and Translation

Congenital diaphragmatic hernia is a multi-genetic pathological condition whereby the processes of branching morphogenesis and embryonic pulmonary vascular development are globally affected with an arrest during the pseudoglandular stage of development. CDH is a birth defect characterized by herniation of abdominal contents into the thorax caused by a defect in the diaphragm. It occurs in two in 10,000 births (12) and presents with a wide range of severities and neonatal outcomes. Management of CDH has undergone a significant paradigm shift changing from a historical surgical emergency to an elective approach after a variable period of stabilization (13).

The morbidity and mortality associated with CDH is the result of a complex interplay between pulmonary hypoplasia and severe pulmonary hypertension in addition to pre-existing factors, including the genetic background of the patient and potential associated malformations. Over the past 35 years, we have witnessed a shift in the survival of babies with CDH (14); however, results are still variable depending on severity of pathology, experience of multidisciplinary teams and efficacy of institutional protocols (15). Moreover, the exponential increase in understanding the natural history of the disease combined with the development of expert fetal intervention centers have also contributed to remarkable outcomes. Nevertheless, outcomes in the most severe cases remain poor despite major advances in prenatal diagnosis, severity prognostication, fetal intervention, and outstanding neonatal intensive care.

#### TO—The Start of a New Era in Management of Diaphragmatic Hernia

Tracheal occlusion is an innovative experimental approach offered to the most severe cases of CDH to drive accelerated lung growth. Hedrick et al. first described TO in 1994, which was referred to as “*Plug the Lung until it grows* (*PLUG*).” In this study, the investigators performed tracheal ligation in 75-day gestation fetal lambs followed by delivery at 130 days. Their results demonstrated an increase in the lung dry weight, DNA, and protein content in the ligated fetal lambs compared to un-ligated lambs (16). Since then, different animal models have been used to study CDH and the associated potential *in utero* therapy including sheep, rabbits, rats, and mice (17–19).

#### Development of Endoscopic TO—The Debut of “Fetendo”

Success of fetal TO relied on a 10–15 cm hysterotomy to gain access to the uterine cavity and the fetus. Exposure of the fetal neck facilitated application of a tracheal clip that obligated removal later on prior to delivery. The group at University of California San Francisco (UCSF) reported their initial experience with PLUG in a 27-week human fetus with CDH. However, the need for this hysterotomy resulted in significant fetal morbidity and mortality secondary to preterm delivery (20). Subsequently, the same group developed a Video-Fetoscopic TO technique (*Fetendo*-PLUG) to circumvent the need for a large hysterotomy (21). This minimally invasive technique was applied successfully in all four fetal lambs, however, only two lambs survived to cesarean section delivery. At necropsy, these lambs demonstrated enlarged lungs and reduced viscera consistent with the expected effects of TO.

#### Michael Harrison’s First Randomized Controlled Trial of Fetal Endoscopic Tracheal Occlusion (FETO) and the Setup of the “Total” Trial

Harrison et al. published the first randomized controlled trial of fetal endoscopic tracheal occlusion in 2003. Women between 22 and 27 weeks carrying fetuses with severe left-sided CDH [liver herniation and lung-head ratio (LHR) <1.4] and with no other anomalies were randomized to either FETO or standard postnatal care (22). However, enrollment in this study was prematurely terminated secondary to the unexpectedly high survival rates seen in the standard postnatal care with 77% of patients surviving to 90 days.

Deprest et al., demonstrated that percutaneous FETO can be performed successfully and is associated with an apparent increase in neonatal survival. They reported that gestational age at birth, pre-existing lung size, ability to remove the balloon prior to birth, and the lung response are predictive outcomes for survival (23).

#### The Current Era of FETO

Advances in endoscopic techniques and preliminary data resulted in adoption of FETO among a handful of highly specialized fetal intervention centers. A recent randomized controlled trial demonstrated a beneficial effect of FETO on survival compared to expectant management (24). These results were very similar to a large multicenter study on severe diaphragmatic hernia treated by FETO where they reported an overall survival rate of 49% in 188 fetuses (25). Furthermore, a recent article demonstrated that FETO is associated with an infant survival rate of 47% in the most severe cases of left-sided CDH with an observed-to-expected LHR of <= 35% (26). Despite the apparent effects of TO and the substantial research demonstrating its local effects on lung growth and peripheral pulmonary circulation, it is currently poorly understood why CDH babies with or without TO have a variable clinical response.

## The Nuts and Bolts of TO

Tracheal occlusion results in accelerated lung growth in animal models with varying effects on type II pneumocytes. Wu et al. demonstrated that following TO in rabbits with a surgically created CDH, the lungs are larger than controls and this was proportional to the duration of TO. Moreover, occluded lungs are comparable to controls morphometrically. Conversely, the density of type II cells was inversely related to the gestational age at which TO was performed. TO performed in rabbits at 26 days was associated with a lower density of type II cells. However, density of type II cells was normal when TO was performed at 28 days gestation (27). Such reduction in the number of type II pneumocytes may be attributed to decreased cell proliferation, increased apoptosis, or augmented differentiation to type I cells. De Paepe et al. suggested that the reduction in type II pneumocyte density following TO may be attributed to an accelerated terminal differentiation to type I cells (28). Furthermore, Roubliova et al. demonstrated that TO has a gestational age-dependent effect on muscularization of intra-acinar (30–40 μm) vessels. TO at 26 days was associated with 85% muscularized vessels relative to normal controls. However, when TO was delayed to 28 days, the number of muscularized vessels decreased significantly (73% less than controls) (29).

## Interplay Between Fetal, Maternal, and Placental Networks Following TO: A Case for Heterogeneous Topological Zones

Most of the contemporary research on TO focuses on isolated changes within the lung without taking into account considerable interplay between the mother, fetus, and fetoplacental dyad. TO is not only expected to produce local changes in the fetal lungs but one should also consider the effect of fetal surgery on mom, the inflammatory and endocrine responses, placental function, mast cells activation, and immune system among others. The dynamic interaction and effect of all of these factors make the case for formation of heterogeneous topological zones in the fetal lungs. The net result of interplay between these factors will dictate the overall clinical response. In this section, we will briefly discuss suggested mechanisms for accelerated fetal lung growth following TO and how these mechanisms may result in the formation of heterogeneous topological zones.

### Fetal Factors: Role of Lung Mechanical Forces

Mechanical forces are a key element in many fundamental processes that operate during development, including cell sorting, differentiation, and compartmentalization (30). Therefore, it is not surprising that mechanical forces not only play a very important role in mechanisms of normal lung development but also an important role in accelerated lung growth following fetal TO.

Fetal pulmonary growth is a classic example of how the mechanisms of mechanosensitivity and mechanotransduction work in concert resulting in cellular proliferation and growth. This phenomenon is dependent on the normal trans-pulmonary pressure gradient generated by lung fluid production and the fixed resistance of the glottis (31) accentuated by FBMs. Sheep studies have demonstrated a rate of fluid secretion by epithelial cells of 3.5–5.5 ml/h/kg resulting in an increase in the luminal pressure 2–3 Torr above amniotic fluid pressure. The resulting trans-pulmonary pressure gradient will actively distend the lung and passively distend the chest wall to a volume equivalent to the functional residual capacity (32, 33). The developmental role of airway distension is the basis of pulmonary hypoplasia seen in some clinical scenarios. In oligohydramnios, decreased amniotic fluid is the reason for the lack of airway distention; however, other scenarios, such as CDH, skeletal dysplasia and neuromuscular paralysis of the diaphragm, and lack of airway distention, are secondary to alterations in the mechanical environment of the thorax (34, 35). Previous research has demonstrated that TO can reverse the pulmonary hypoplasia observed in various experimental and pathological conditions, including severe oligohydramnios, bilateral nephrectomy, and CDH (36, 37).

Mechanotransduction is defined as the functional link between sensing of mechanical stimuli and the resultant generation of a biochemical response. This concept builds on the fascinating viscoelastic cellular properties that straddle characteristics of both solids and liquids. Upon application of force, cells will deform in a temporal manner and will return to their initial state once that stimulus is removed (38). Therefore, cellular deformations and the accompanying alterations in cellular–extracellular matrix interactions are the hallmarks of mechanotransduction (39). This mechanical behavior of tissues is defined by an interconnected network of cell–cell junctions, cell–matrix adhesions, intracellular filament networks, and extracellular matrix.

Cells in the developing lungs are subjected to different forces, including tensile, compressive, hydrostatic, and fluid shear stress. The translation of local extrinsic mechanical events into fast and long-standing changes of cellular phenotype is dependent upon the function of a complex cellular network of molecules and structures which are capable of sensing and responding to mechanical force. This force-sensing can occur through force-induced conformational or organizational changes in cellular molecules or structures (40). Some structural cellular elements involved in sensing and integration of mechanical forces include stretch-sensitive ion channels (41), cadherin complexes in cell–cell adhesions (42), G protein-coupled and tyrosine kinase receptors (43), and integrins (44). When the mechanical signal is received, the signal is amplified and propagated through activation of intracellular signaling pathways, which involves focal adhesion kinase, Rho-family GTPases, mitogen-activated protein kinase-extracellular signal-regulated kinase (MAPK-ERK), and others (40).

Physical properties of the ECM play a very important role in fetal development by directing the differentiation of stem cells to specific lineages (45). As cells differentiate, they generally become stiffer in a cell-type-specific manner and further tune their stiffness to the properties of the local materials that make up their ECM, progressively stiffening on more rigid material (46). The changes in cell stiffness can in turn directly affect mechanotransduction. Stiff-differentiated cells deform less than compliant stem cells in response to applied mechanical stress, resulting in attenuated level of mechanotransduction (46). This specificity feature of mechanotransduction allows undifferentiated cells in the embryo to respond in a highly sensitive manner to mechanical stimulation, while differentiated cells are less sensitive to acute mechanical perturbations in homeostatic tissues (38). The aforementioned physical factors underscore why fetal lungs can respond more to mechanotransduction and the accompanying changes in the ECM than mature differentiated tissues.

Taking into account that mechanical forces are derived from the collective relationship between cells; between the cells and extracellular matrix and also between cell–cell junctions and cell–matrix adhesion and intracellular filament network, there is 3-dimensional (3-D) distribution of tension and gradient of mechanical stress in tissues. As a result, the sites of mechanotransduction depend on the spatial connectivity and material properties of the tissue network (47, 48). We propose that changing lung tissue 3-D architecture after TO will change distribution of tension and gradient of mechanical stress in the lung, cell connectivity and shape, local sites of mechanotransduction, topology of cell membrane receptors, ionic channels and signaling complexes, matrix remodeling, and alteration of protein expression in the cells with subsequent adjustment of cellular function. In other words, changing lung tissue geometry after TO might have a significant effect on the global function and structure of the developing lung, including sensitivity and response to different stimuli.

However, hydrostatic pressure generated in the airway following TO, 3-D distribution of tension and the gradient of tissue mechanical stress may not be uniformly distributed to all lung zones. Therefore, we hypothesize that TO results in a variable 3-D geometry in different lung zones resulting in a heterogeneous response. Moreover, it is also important to note that the mechanical stability of proteins and, therefore, mechano-sensing can be tuned by basic biological mechanisms, such as pH, ionic strength, and redox state. For example, redox state can regulate stability of proteins involved in mechano-sensing by regulation of the disulfide bond formation (47). Therefore, we also suggest that a change in any or all of the following parameters (redox state, pH, and ionic strength) in the “fetal lung environment” after TO and maternal fetal surgery could tune mechano-sensing, direct lung development and may potentially be involved in the formation of heterogeneous topological zones.

### Fluid Accumulation and Mechanical Forces Change in the Fetal Lung after TO

An elegant study by Papadakis et al. demonstrated that in comparison to TO, fetal sheep where the tracheal fluid was substituted by an equal volume of saline did not have the same amount of lung growth despite a constant intratracheal pressure between 3 and 5 Torr (49). Whether this finding is due to tracheal fluid growth factors is still unknown. Could there be a role for the biological natural lung fluid on the viscoelastic properties of lung tissue? Also, does the viscosity of the mucus secreted by the airway play a significant role in that process? Moreover, as discussed later, could changes in fetal lung microbiome also explain some of the variable changes in pulmonary growth in relation to saline versus natural lung fluid? The researchers in the former study demonstrated that following tracheal ligation, there is an abrupt increase in the net lung fluid production of up to threefold. However, overall net lung fluid production seems to decrease relative to pre-ligation status. Such phenomenon may be attributed to either reduced secretion or accelerated reabsorption mediated by cortisol or catecholamine surge following surgical stress similar to the peripartum state (50). Surprisingly, net lung fluid production increases after 3 days possibly secondary to increased secretory cell mass attributed to cell proliferation. Other investigators similarly reported that two-thirds of the increase in pulmonary DNA content following tracheal ligation in fetal sheep occurs between days 2 and 7 (51). Moreover, in another study, lung growth after TO demonstrated a lag of 3 days prior to significant changes in both lung-body weight ratio and radial alveolar counts (28). Such phenomenon may be due to the time needed to allow for accumulation of enough bronchial tree fluid to sufficiently change the 3-D geometry of fetal lung tissue, therefore, stimulating mechanotransduction.

### Dynamic Changes in FBMs

Fetal breathing movements are one of the potential physical forces that stretches the developing airspaces and results in mechanotransduction with subsequent lung growth. FBMs can be noticed as early as 8 weeks of gestation (end of the pseudoglandular stage) and are used to assess fetal wellbeing (5). Fetal TO will result in accumulation of lung fluid beyond the site of occlusion resulting in an increase in the trans-pulmonary pressure. However, it is unknown whether such changes will be associated with alteration in the FBMs. Experimental studies aiming at interruption of FBM, whether *via* a fetal cervical spinal cord transection above the phrenic nucleus or increase in chest wall compliance, have demonstrated a decrease in lung growth, lung tissue DNA content, cell proliferation, and cell survival (52, 53). In contrast to cyclical stretch induced by FBM, TO results in sustained mechanical stretch without an increase in the mRNA expression or protein levels of matrix metalloproteinases (MMPs) (54). During FBM, there is laminar flow of fluid distributed across most regions of the developing bronchial tree. Such laminar flow may recapitulate the same effects of laminar flow on endothelial cell survival in the vascular tree (55, 56).

### The Role of Periodic Agitation of Lung Tissue Induced by Cardiac Contractions on Lung Development

All mechanical forces in the developing fetal lungs will interact with the periodic cardiac mechanical oscillation. The role of periodic cardiac-dependent mechanical stimulation of the fetal lung in normal pulmonary development is vastly unexplored. Although we would expect a change in cardiac-dependent mechanical stimulation of the developing lung in CDH and following fetal TO, the relationship is unknown. We propose that the increased distension of lung secondary to fluid secretion in the closed bronchial tree following TO will result in a temporal change in the cardiac-dependent mechanical stimulation of the lung. Moreover, such stimulation may have substantial influence on lung development.

The heart as an electro-mechanical oscillator might play an important role not only in fetal lung development but also possibly in the coordination and synchronization of common mechanisms of embryonic 3-D organization and fetal development. One possible way for cardiac-dependent orchestration of lung development might be synchronization of the internal oscillators by the electro-magnetic field generated by the heart. The coupled oscillators are known to be a vigorous system resistant to both internal and external noise (57). We suggest that cardiac level synchronization may also work on the level of metabolic synchronization in different organs and tissues.

In addition to the spatial and temporal three dimensions, living systems may also demonstrate a self-generated “electrical dimension” (58). The electrical field generated by the heart may have an important role in generation of an electrical dimension through the body and can play a significant role in lung and fetal development. It has been shown that the electric field generated by the heart in embryos has an impact on cardiomyocyte morphology and cardiac organogenesis (59). These electrical effects might be mediated not only by changing cellular polarization but also redistribution of receptors in the cell membrane by electric fields (59) but will this electrical field produce similar effects on lung cells?

Indeed, the role of periodic cardiac-dependent mechanical stimulation of fetal lung and the role of electro-magnetic oscillation of heart is interesting not only for understanding cardiac-dependent modulation of lung growth mechanisms after fetal TO but also important for understanding the basic principles of organization of mammalian living systems.

### Additional Fetal Factors that May Play a Role in Fetal Lung Growth following TO

#### Role of Fetal Lung Tissue Injury

In addition to the previously discussed factors, TO is a form of fetal and local lung injury that will certainly play an important role in the associated lung growth response. Not only is there a surgical insult manifested by fetal injury and TO, but also there is the accompanying role of fetal lung fluid accumulation. Lung fluid accumulation in the occluded fetal airway may play a dual role in the process of lung growth. First, it will result in mechanical stimulation of lung growth and, second, it may constitute some form of local tissue stress/damage. Both of these mechanisms may be variable and contribute to the formation of heterogeneous topological zones.

#### The Role of Activation of Mucosal Mast Cells and Damage-Associated Molecular Pattern Substances (DAMPs)

We believe that lung mast cells could be an important sensor of pressure-induced damage of lung tissues. These cells may potentially sense excessive mechanical distension of lung tissue and subsequently respond to such stimulation. In this way, mechanical stress may represent a priming signal for mast cell activation. However, it is currently unknown how lung mast cells respond to mechanical stress after TO. We postulate that in addition to the previously mentioned mechanism, an alternative pathway for involvement of lung mast cells in the modulation of accelerated fetal lung growth following TO is activation by DAMPs and biologically active products formed during activation of the complement and blood coagulation systems.

The number and maturity of mast cells increase during embryonic development (60, 61), and because mast cells express toll-like receptors, including TLR2, TLR3, TLR4, TLR6, TLR7, and TLR9, they have the ability to sense some DAMPs (62). Moreover, recent studies suggest that mast cells have an important role in regulating the fetal inflammatory response. For example, they are responsible for the transition from scarless fetal wound healing without inflammation observed at the early stages of fetal development to healing with inflammation and scar formation at the later stages (61).

Out of all potential mast cell products, mast cell proteases (tryptase, chymase, cathepsin G) could possibly be critically important in the regulation of fetal lung growth following TO. This is based on the broad-spectrum activity these proteases possess, including activation of pro-MMP-9 by chymase; generation of angiotensin II by chymase and Cathepsin G independent of angiotensin-converting enzyme; inactivation of cytokines/chemokines by chymase and cathepsin G; processing “big” endothelin by chymase; and activation of protease-activated receptor (PAR)-2 by tryptase (63–65). In addition, mast cell proteases may generate the anaphylatoxins C3a and C5a by direct proteolysis of C3 and C5 complement proteins (66, 67). In addition to the aforementioned role, mast cells can also recruit different cells and systems, for example, the blood coagulation system and leukocytes, for modulation of lung growth. These data suggest that mast cells may be an important participant in regulating fetal lung growth after TO.

#### The Possible Role of Local Sterile Low-Grade pulmonary Inflammation in Modulating Accelerated Lung Growth following Fetal TO

Increasing the hydrostatic pressure in the fetal bronchial tree following TO will lead to cellular stress in some topological areas of the lung where there is a more significant pressure gradient. Such cellular stress/damage occurs because of either direct action of increased physical forces on cells or secondary to the action of biologically active substances released from damaged and activated cells or formed during activation of triggered systems, such as blood coagulation, fibrinolysis, and complement systems. As a result, cellular stress/damage will produce an increased amount of DAMPs (68–70).

Release of DAMPs after fetal TO may be either due to passive secretion into the extracellular environment; cellular death; active release as a result of cellular stress or cell activation; or the result of formation of extracellular matrix damaged fragments. Heat shock proteins (HSP60, 70, 90), high-mobility group box 1, mitochondrial transcriptional factor A, extracellular ATP, IL-1β, TNF-α, ROS, ROS-modified molecules, extracellular-matrix breakdown products, and angiotensin II may work as DAMPs and some of these DAMPs can interact with toll-like receptors in fetal lung after fetal TO.

But how does this damage (tissue stress) and the subsequent local sterile low-grade inflammation interact with the mechanisms of accelerated fetal lung growth induced by mechanical forces? The alteration of lung homeostasis combined with the damage and controlled inflammation may have multiple effects on the mechanisms that control lung development during accelerated lung growth induced by TO. Notably, these effects might have significant differences in different topological zones of fetal lung depending on the different extent of local tissue stress and injury induced by mechanical forces.

It is well known that antenatal inflammation can modulate maturation and development of the fetal lung and the immune system. Intra-amniotic injection of IL-1 induces lung maturation in fetal rabbits and sheep mostly by increasing levels of surfactant protein mRNAs, increasing levels of saturated phosphatidylcholine and improving lung compliance (71, 72). The lung maturational effects also occur when IL-1 or endotoxin is given by fetal intratracheal instillation (73). Nevertheless, inflammation (experimentally induced by intra-amniotic injection of LPS and IL-1 or induced by fetal tracheal instillation of LPS and IL-1) can also affect expression of growth factors (transforming growth factor-beta, fibroblast growth factor-10, connective tissue growth factor, bone morphogenetic protein-4) and cause structural changes in the fetal lung, such as alveolar and microvascular simplification (74). Intra-amniotic injection of LPS in sheep decreases alveolar number, causes thinning of the alveolar septae, and increases the size of the alveoli (75). Also, LPS injection in sheep fetal lungs inhibits some genes involved in vascular development, including VEGF-A, VEGFR2, and NOSIII, and causes smooth muscle hypertrophy in arterioles in addition to adventitial fibroblast proliferation (76). Moreover, other growth factors may be involved in modification of the vascular wall. For instance, bFGF can alter the function of endothelial cells, change the phenotypic plasticity of vascular smooth muscle cells and stimulate their proliferation (77–79). Despite such information, the mechanisms of alteration of lung development pathways by intra-amniotic inflammation are still poorly understood.

All reviewed effects of inflammation on fetal lung development are related to quite a strong inflammatory response induced by LPS or IL-1. However, we propose that the effects of local sterile low-grade inflammation that occurs in TO might be different, as it will be more targeted and present in limited topological sites of lung tissues as previously discussed. The potential role of local inflammation in regulation of fetal lung growth after TO may include modulation of the mechanisms of communication involved in lung growth, metabolism, and remodeling of the extracellular matrix.

#### Do the Lung Inflammasomes Participate in Modulation of Lung Growth following TO?

Traditionally, it has been postulated that inflammasomes, mostly expressed in myeloid cells, act as intracellular multiprotein complexes primarily involved in the host’s innate immune system response to exogenous infectious microbes, non-infectious toxic environmental agents, and endogenous danger signals (80, 81). The key effector function of inflammasomes is to generate the maturation of pro-inflammatory cytokines IL-1β and IL-18 *via* caspase-1 (82). However, recent evidence suggests that inflammasomes are involved in regulation of many cellular responses, including metabolism, proliferation, autophagy, and apoptosis (83). Besides myeloid cells, inflammasomes are also expressed in other cell types, such as cardiac fibroblasts and endothelial cells (84–86). Activation of inflammasomes in the lung endothelial and epithelial cells could, for example, transiently change the phenotype of these cells along with their interactions with other lung cells.

A potentially new intriguing way for participation of inflammasomes in TO-mediated lung growth depends on their ability to possibly monitor cellular perturbation (“homeostasis-altering molecular processes”), for example, by NLRP3 inflammasome (87). Potential stimuli for activating NLRP3 in such mechanism may include low cellular potassium level, amino acid starvation, and reduced fatty acid oxidation (87). We, therefore, hypothesize that monitoring of “homeostasis-altering molecular processes” by NLRP3 inflammasome may be beneficial in the setting of modulation of fetal lung growth following TO because of cellular stress.

#### Possible Role of the Complement System

The complement system can also participate in the modulation of fetal lung growth after TO. Recent evidence demonstrates that complement components C3 and C5 and anaphylatoxins C3a and C5a are involved in the processes of cell reprogramming, cell lineage regulation and regulation of stem-cell phenotype and these effects depend on local tissue production of complement proteins (88). We have previously discussed that mast cell proteases can generate C3a and C5a anaphylatoxins by direct proteolysis of the C3 and C5 components of complement. These anaphylatoxins interact with C3aR and C5aR1 or C5aR2 receptors present on different cells resulting in large spectrum biological responses (89). Therefore, some of the cellular responses to the action of anaphylatoxins, such as cell activation, change of phenotype, and increase production of ROS could be involved in the mechanisms of fetal lung growth after TO.

However, a more interesting participation of the complement system in accelerated lung growth may be on the level of the so-called intracellular complement system. Recently, it was shown that intracellular complement activation has an important role in regulation of some of the basic cellular processes, such as metabolism and NLRP3 inflammasome activation (90, 91). Therefore, we hypothesize that the cellular stress in fetal lungs after TO could lead to alteration of the intracellular function of the complement system resulting in disruption of the complement-dependent mechanisms regulating NLRP3 inflammasome activation and cell metabolism.

#### Amniotic Fluid Replacement and the Possible Change in Fetal Microbiome and Setup of the Basal Level of Activation of the Humoral Trigger Systems

The long-standing hypothesis that a fetus develops in a sterile environment and is born with a sterile gut is being challenged (92). Recent evidence in animals and humans show that prenatal transfer of bacteria from mother to fetus may exist (93). The fetal intestinal tract is normally bathed in amniotic fluid that has been swallowed. If amniotic fluid contains bacteria they will be transferred to the intestine and lung. The gut colonization in the fetus should play a very important role in development of the immune system and also in setting up the basal level activity of blood coagulation and complement systems. We speculate that fetal surgery and escape of some of the amniotic fluid during fetal TO with replacement by Ringer’s lactate solution can be a reason for the temporary change in the fetal microbiome in the gut. Further research is warranted to examine whether such potential microbiome change will play some biologically important role in lung development in the short and long terms?

The challenge in discussing the suggested mechanisms involved in accelerated fetal lung growth after TO is whether these signals/mechanisms will work in a uniform fashion in different zones of the lung to produce a homogeneous landscape. We suggest that the action of these mechanisms, their interactions, and intercommunications will produce different topological zones in the fetal lung. The key for understanding such non-uniform topology in the lung is the differences in the complex interaction between all factors involved in accelerated lung growth, for example, the dissimilar degree of hydrostatic pressure in the bronchial tree with a variable 3-D geometry, inconsistent cellular stress/damage with the consequent local sterile inflammation, and the variable lung mast cells response among others.

## Maternal Factors: Role of Placenta in Lung Growth Following TO

The human placenta is a unique, transient organ that serves as the primary interface between the maternal and fetal circulations. Throughout pregnancy, the placenta provides nutrition, gas exchange, waste removal, and also serves as a source of hematopoetic stem cells and endocrine and immune support for the developing fetus, as well as the site of molecular exchange between the maternal and fetal systems (94). The accelerated fetal lung growth after TO will lead to an increased demand in nutrients to support such growth. Undoubtedly, this should change the demand on the placenta. But how does the placenta sense the increased demand for nutrients? There is evidence that the mTOR pathway in the placenta functions as a nutrient sensing mechanism (95). According to this hypothesis, mTOR integrates nutrient and growth factor signaling to control nutrient transport from mother to the fetus. However, the adaptation of such function in accelerated lung growth is currently unknown.

## Maternal and Fetal Systemic Factors and Lung Development Following TO

**Figure 1:**
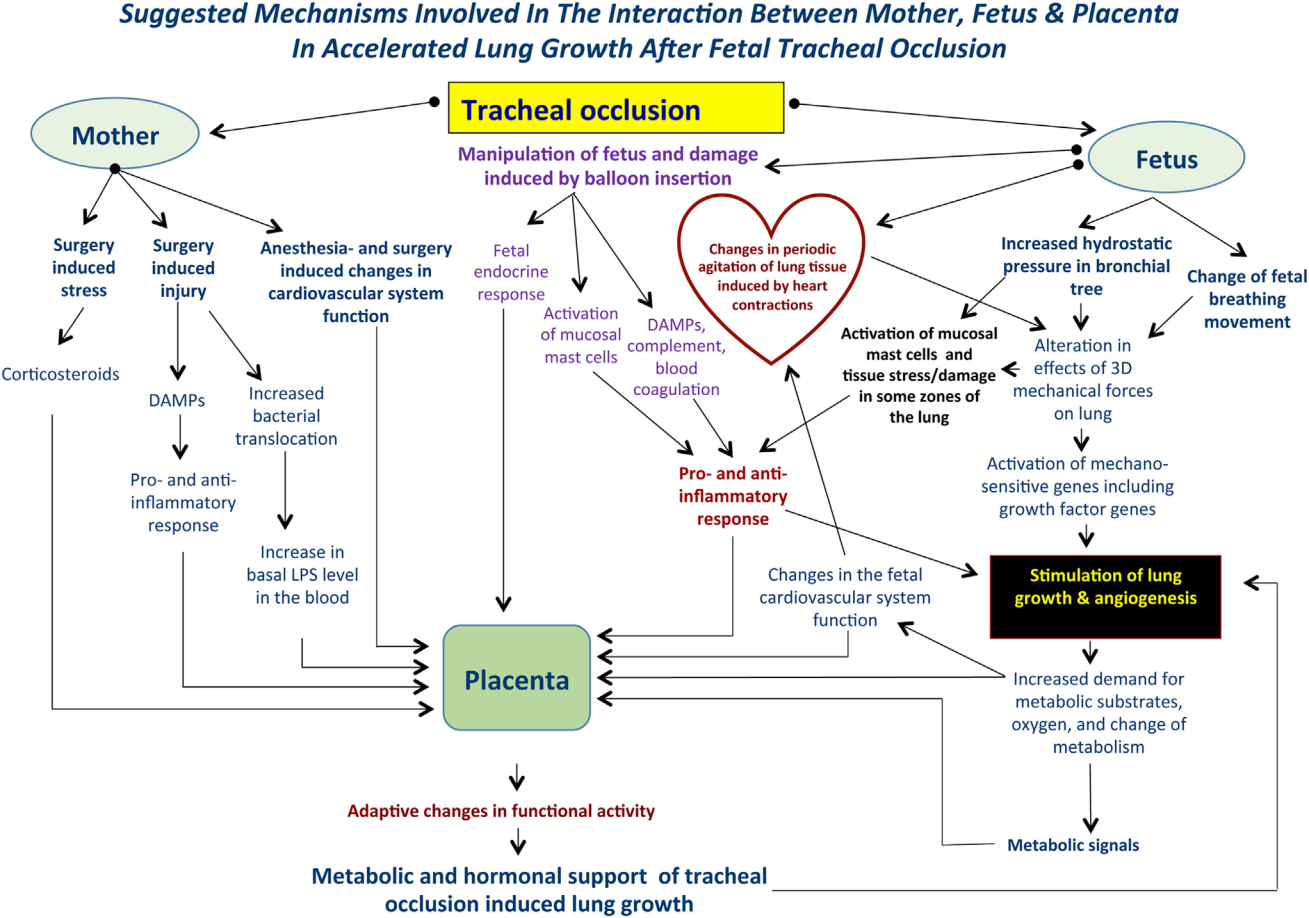
Suggested mechanisms involved in the interaction between mother, fetus, and placenta following tracheal occlusion (TO). TO results in an increase in the tracheobronchial tree hydrostatic pressure that will be accompanied by an alteration in the 3-dimensional geometry of local tissues. Ultimately, these changes result in activation of the mechanosensitive genes and stimulation of lung growth and angiogenesis. At the same time, TO may activate lung mast cells and release damage-associated molecular pattern substances (DAMPs) with a subsequent pro- and anti-inflammatory response. Such complex mechanisms are functioning within a background of maternal fetal surgery involving surgical stress, injury, possible bacterial translocation, and anesthesia induced changes in the cardiovascular system. Local changes in the fetal lung will result in an increased demand for metabolic substrates and provide the signals for altering placental function. Moreover, maternal and fetal systemic effects will also result in modulation of the placental function providing the metabolic and hormonal milieu to support accelerated lung growth. These effects are unique to each case.

The global picture of accelerated lung growth following TO will become more complicated if we take into account some systemic factors, including preexistent maternal stress, surgical stress; fetal stress induced by anesthesia and surgery; systemic effects of narcotics; fetal response to pain (surgery, balloon insertion, and inflation inside the trachea), maternal and fetal endocrine response, and the genetic background of fetuses with CDH. Moreover, will fetal surgery induce an increase in maternal bacterial translocation resulting in an increased basal level of endotoxin in maternal blood? And will the increased bacterial translocation have some influence on fetal lung development after TO? (Figure 1).

### Metabolism—An Organizing Noise in Lung Development after TO

Commensurate to the dynamic processes involved in pulmonary development and growth, metabolism mirrors these active processes and is constantly changing to match the ever-increasing needs of the lung, including proliferation, differentiation, and biosynthetic demands. Rapid cellular growth and proliferation needs large amounts of individual building blocks, including amino acids and nucleotides. Therefore, cells preparing for proliferation are required to utilize a unique repertoire of metabolic processes with a change to a “Warburg-like metabolism” which is dependent on aerobic glycolysis to achieve growth (96). In contrast to conventional glycolysis, actively growing cells will shift the glycolytic flux toward lactate production thus supporting the pentose phosphate shunt by generating large amounts of the reduced form of nicotinaminde adenine dinucleotide (NADH). This process allows for both adequate ATP production and a surplus of carbon and NADH for growth (97). Furthermore, to effect growth, tissues need to acquire a unique metabolic state that is achieved *via* synergism between aerobic glycolysis and glutaminolysis resulting in a shift to glutamine use instead of pyruvate to supply the tricarboxylic acid (TCA) cycle (98).

#### The Interplay between Metabolism and Angiogenesis: Glycolysis Is the Crux of Endothelial Cell Metabolism

Endothelial cells do not rely on mitochondrial respiration for energy production but rather utilize glycolysis that is surprising considering the obvious inherent advantage of being within the high oxygen content blood stream within the blood vessels (99). This is in the face of reduced ATP production *via* glycolysis (2 mol of ATP per mole of glucose) in comparison to 36 mol of ATP per mole of glucose in mitochondrial respiration. Several researchers have highlighted some potential reasons and advantages for this fascinating phenomenon, including the ability to sprout into hypoxic environments, thus allowing endothelial cells to provide oxygen to ischemic tissues (100–102), generation of less reactive oxygen species secondary to synthesis of NADH *via* the pentose phosphate pathway (103), faster ATP production will enable the endothelial cells to change from their “quiescent” state into a “tip” position (104) and will promote biomass production *via* the pentose phosphate and serine biosynthesis pathways, which are glycolytic side pathways (105).

Glycolysis is responsible for generation of up to 85% of endothelial cell ATP (106). In order to match the dynamic requirements of angiogenesis, endothelial cells are very plastic and have the ability to switch from their “quiescent” to “sprouting” state upon need. Such remarkable ability depends on adjustment of the endothelial cell metabolism. However, the mechanisms involved are not yet fully understood. Wilhelm et al. in 2016 demonstrated that Forkhead box protein O1 acts as a gatekeeper of endothelial quiescence *via* suppression of c-MYC and reduction of glycolysis (107).

6-Phosphofructo-2-kinase/2,6-bisphosphatase-3 (PFKFB-3) is crucial for endothelial cell-mediated metabolism and motility. PFKFB-3 stimulates glycolysis by the constant supply of fructose-2,6-bisphosphate, which is a potent activator of the glycolytic rate limiting enzyme phosphofructokinase-1 (PFK-1) (106). Moreover, PFKFB-3 promotes tip cell phenotype by relocating to the lamellipodia and filopodia of migrating endothelial cells where they generate spatially focused high levels of ATP needed for actin–myosin contraction during vessel sprouting (108). Once sprouting occurs and endothelial cells achieve contact with neighboring cells, they revert to their “quiescent” state whereby PFKFB-3 relocates to the perinuclear cytosol (106). Moreover, blood flow through the newly established network results in endothelial cell shear stress resulting in krueppel-like factor 2-dependent transcriptional suppression of PFKFB-3, hexokinase 2, and PFK-1, thus reducing glucose consumption and glycolysis (109).

**Figure 2:**
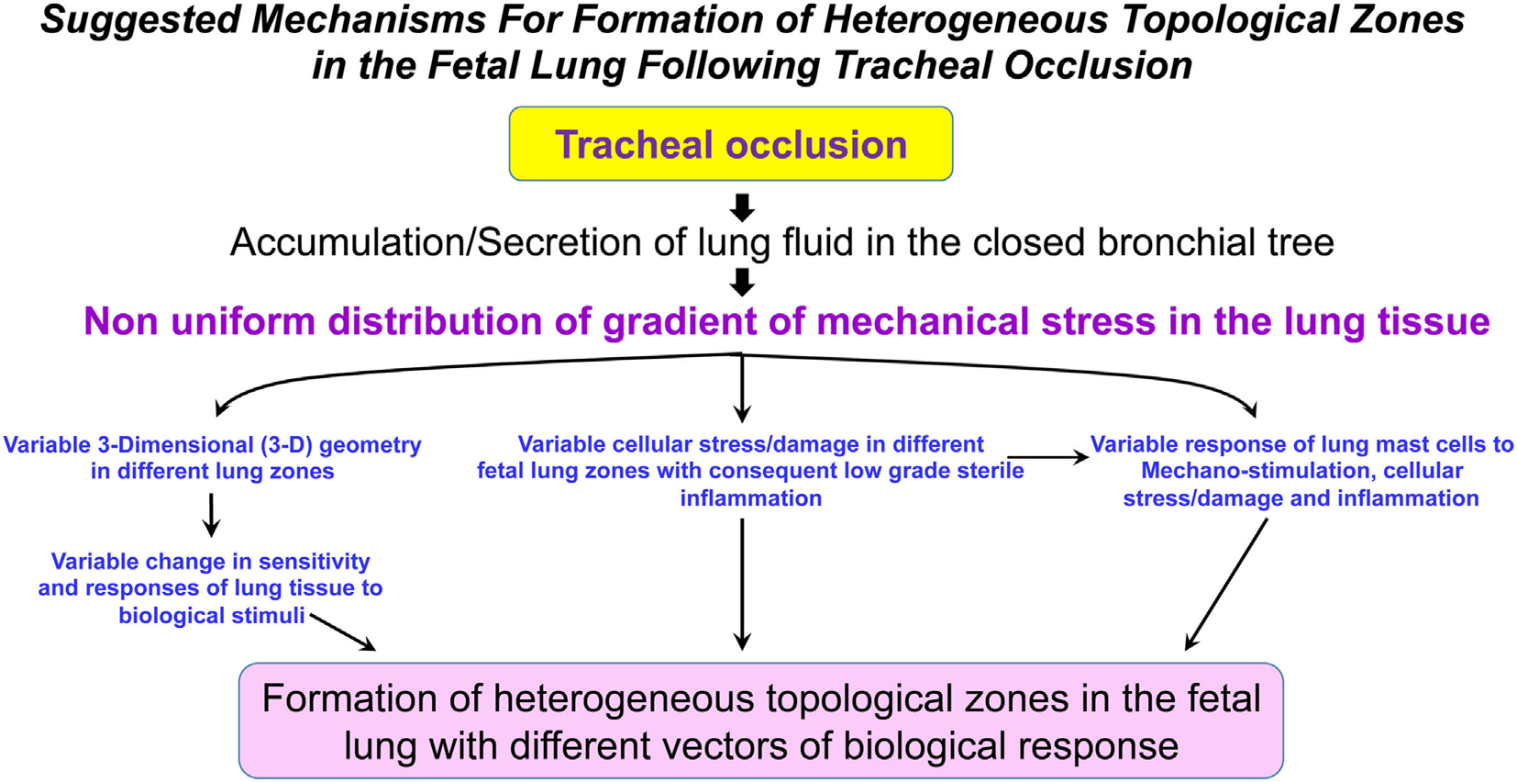
Suggested mechanisms for formation of heterogeneous topological zones in the fetal lung following tracheal occlusion (TO). We propose that TO results in a non-uniform distribution of the mechanical stress gradient in the fetal lungs. This non-uniform mechanical stress will be accompanied by variable changes in the different lung zones with respect to 3-D geometry, cellular stress/damage, and lung mast cell response. Ultimately, the interplay between these factors will result in formation of heterogeneous topological zones in fetal lungs with different vectors of biological response.

It is currently unknown whether TO will result in a homogeneous shift in the metabolic landscape of the fetal lungs. The reason for this question arises from our previous discussion regarding the numerous factors involved in the process of accelerated lung growth following TO (mechanical stimulation, tissue stress, tissue damage, local sterile low-grade inflammation, maternal and fetal systemic factors, change in the fetal microbiome, among others). The combined action of these factors could be unique in different fetal lung zones and may lead to a non-uniform change in the metabolism of fetal lungs (Figure 2). Furthermore, it is currently unknown how metabolic changes in different lung zones affect angiogenesis/vasculogenesis.

## Conclusion: Are There Variable Topological Zones in Fetal Lungs after TO?

We suggest that the key principle in understanding changes in fetal lungs after TO is taking into account formation of variable topological zones in the fetal lungs. Temporarily the lungs will look like a dynamically changing topologic mosaic with varying proliferation rates, dissimilar scale of vasculogenesis, different patterns of lung tissue damage, diverse metabolic landscape, and different organization of the newly formed lung tissue with normal and abnormal structures. The reasons for this dynamic topological mosaic pattern in fetal lungs may include the distinct degree of increased hydrostatic pressure in different parts of the lung, divergent pattern of physical characteristics of three dimensional mechanical stress, dissimilar degree of lung tissue damage and responses to this damage, and incomparable patterns of altered lung zones with variable response to systemic maternal and fetal factors, among others. In this scenario, the fetal lung will form zones with different cellular environment, similar to the case of stem-cell niche. The local interaction between different cells, signals, and systems might lead to the formation of a common vector of biological response unique to this local zone. However, the common vector of lung tissue response in the adjacent topological zones might be slightly different.

Therefore, could we discuss common changes in the fetal lung after TO without taking topological differences of lung zones into account? In our opinion, this is incorrect. For example, if we discuss the possible role of inflammasomes in mechanisms of accelerated fetal lung growth, we need to consider that, in some lung zones, there may be activation of inflammasomes in myeloid cells with production of IL-1; but, in other zones, there may be activation of inflammasomes in endothelial cells with changing phenotype and vasculogenesis. The same situation exists for metabolism; the large increase in glycolysis compared to the TCA cycle could be in the zones with significant growth. On the other hand, some balance may exist between the level of glycolysis and TCA cycle in other zones, without significant growth. In addition, discussion of all the potential mechanisms of accelerated fetal lung growth following TO needs to take into account that hypoxia, acidic environment, incomplete maturation of cells, and physiological systems constitute a fundamental background where these mechanisms work.

Much work is still needed to uncover the hidden mechanisms of accelerated fetal lung growth following fetal TO. Future experiments will have to determine how different factors, signals, and systems interact with each other to delineate a hierarchy of communicating mechanisms involved in signaling, extracellular-matrix remodeling, cell sorting, and differentiation and compartmentalization during accelerated fetal lung growth induced by fetal TO. This will also bring us closer to an understanding of the biological phenomenon of—how apparent “chaos” in biological systems with an interplay of signals and participants finally convert to a fine “order.” A clear understanding of how different systems (mechanical stimulation, tissue stress, tissue damage, local sterile low-grade inflammation, maternal and fetal systemic factors, and change in the fetal microbiome, among others) become organized for working together to produce fine tuning of fetal lung development will lead to new future clinical methods for more effective treatment of conditions with pulmonary hypoplasia, including CDH, giant omphaloceles, following major lung resections and other clinical forms of bronchopulmonary dysplasia.

## Author Contributions

Substantial contributions to the conception of the work. Drafting the work and revising it critically for important intellectual content. Final approval of the version to be published. Agreement to be accountable for all aspects of the work.

## Conflict of Interest Statement

The authors declare that the research was conducted in the absence of any commercial or financial relationships that could be construed as a potential conflict of interest.
